# The effect of low-intensity pulsed ultrasound (LIPUS) for the treatment of upper and lower extremity non-union compared to surgical intervention (PiNGUin): study protocol for a national, prospective, randomized controlled, open-label, multicenter, non-inferiority study

**DOI:** 10.1186/s13063-025-09212-y

**Published:** 2025-10-27

**Authors:** Eva Simone Steinhausen, Andreas Stang, Anika Hüsing, Eva-Maria Huessler, Marcus Brinkmann, Bernadette Schröder, Verena Schröder, Hannah Hohberg-Piel, Marcel Dudda

**Affiliations:** 1 Department of Trauma, Hand and Reconstructive Surgery, University Hospital Essen, Hufelandstr. 55, 45147 Essen, Germany; 2 Department of Orthopedics and Trauma Surgery, BG Klinikum Duisburg gGmbH, Großenbaumer Allee 250, 47249 Duisburg, Germany; 3 Institute for Medical Informatics, Biometry and Epidemiology, University Hospital Essen, Hufelandstr. 55, 45147 Essen, Germany; 4 Institute for Medical Informatics, Biometry and Epidemiology/Center for Clinical Trials, University Hospital Essen, Hufelandstr. 55, 45147 Essen, Germany

**Keywords:** Non-union, Low-intensity pulsed ultrasound (LIPUS), Medical device, Fracture healing, Randomized controlled trial (RCT), Non-inferiority study

## Abstract

**Background:**

Non-unions, defined as fractures not healed after at least 6 months, are a multifactorial complication occurring in 2–10% of all fractures and in up to 30% of patients with certain risk factors. Treatment costs are markedly higher than those associated with normal fracture healing and patients often suffer from pain and loss of function. Surgical revision is the gold standard for treatment; however, the disadvantages are re-hospitalization, possible secondary diseases and side effects such as post-operative infections, complications of anesthesia and embolisms.

Low-intensity pulsed ultrasound (LIPUS) is a low-risk procedure, with skin reactions being the only side effect experienced by a very small number of patients. Patients can use the treatment independently at home, eliminating the need for repeated hospital visits. This study aims to show that LIPUS treatment is not inferior to surgical intervention by a threshold of -25 percentage points in terms of fracture healing in non-unions of the upper or lower extremity.

**Methods:**

PiNGUin will be a five-year national, prospective, controlled, randomized, open-label, multicenter study with a medical device. Patients are randomized into one of two groups: patients in the intervention group will receive daily 20-min LIPUS treatment for 200 days or undergo standard surgical intervention in the control group. Follow-up for both groups will be 12 months. The primary endpoint is fracture healing 12 months after treatment start, defined as bony consolidation of at least 3 out of 4 cortices and functional recovery. Secondary endpoints include time to fracture healing and to full weight bearing, incidence and intensity of pain, functional complaints/activities of daily living, health-related quality of life, complications, adverse events, re-operations or re-osteosyntheses and (re-) hospitalizations, hospital stays, duration and reasons for inability to work. With a one-sided significance level of 2.5%, a power of 90% and an assumed drop-out rate of 10% 184 patients will be required per treatment group.

**Discussion:**

As LIPUS is a low-complication procedure with good results to date, this treatment is considered a potential alternative to surgery. If non-inferiority is proven, patients will have a choice of both invasive and non-invasive procedures in the future.

**Trial registration:**

DRKS00034357 (German Clinical Trials Register, registered March 11th, 2025).

## Administrative information

Note: the numbers in curly brackets in this protocol refer to SPIRIT checklist item numbers. The order of the items has been modified to group similar items (see http://www.equator-network.org/reporting-guidelines/spirit-2013-statement-defining-standard-protocol-items-for-clinical-trials/).

**Table:** 

Title {1}	National, prospective, randomized controlled, open-label, multicenter, non-inferiority study on the effect of low-intensity pulsed ultrasound (LIPUS) for the treatment of upper and lower extremity non-union compared to surgical intervention (PiNGUin)
Trial registration {2a and 2b}.	German Clinical Trials Register (Deutsches Register Klinischer Studien; DRKS): DRKS00034357, registered on March 11th, 2025 (https://drks.de/search/de/trial/DRKS00034357/details)
Protocol version {3}	Version 2.1 of 30–01–2025
Funding {4}	This study is funded by the Joint Federal Committee (Gemeinsamer Bundesausschuss (G-BA))
Author details {5a}	E.S. Steinhausen: BG Klinikum Duisburg gGmbH, Department of Orthopedics and Trauma Surgery, Germany, and University Hospital Essen, Department of Trauma, Hand and Reconstructive Surgery, University of Duisburg-Essen, Germany M. Dudda: University Hospital Essen, Department of Trauma, Hand and Reconstructive Surgery, Germany and BG Klinkum Duisburg gGmbH, Department of Orthopedics and Trauma Surgery, Germany A. Stang: University Hospital Essen, Institute for Medical Informatics, Biometry and Epidemiology, Germany M. Brinkmann: University Hospital Essen, Institute for Medical Informatics, Biometry and Epidemiology/Center for Clinical Trials, Germany A. Hüsing: University Hospital Essen, Institute for Medical Informatics, Biometry and Epidemiology, Germany E.-M. Huessler: University Hospital Essen, Institute for Medical Informatics, Biometry and Epidemiology, Germany B. Schröder: University Hospital Essen, Institute for Medical Informatics, Biometry and Epidemiology/Center for Clinical Trials, Germany V. Schröder: University Hospital Essen, Institute for Medical Informatics, Biometry and Epidemiology/Center for Clinical Trials, Germany H. Hohberg-Piel: University Hospital Essen, Institute for Medical Informatics, Biometry and Epidemiology/Center for Clinical Trials, Germany
Name and contact information for the trial sponsor {5b}	Consortium of: University Hospital Essen AöR Hufelandstr. 55 45147 Essen BG Klinikum Duisburg gGmbH Großenbaumer Allee 250 47249 Duisburg Principle Investigator: PD Dr. med. Eva Simone Steinhausen Department of Orthopedics and Trauma Surgery BG Klinikum Duisburg gGmbH Großenbaumer Allee 250 47249 Duisburg Phone: + 49 203 7688–3123 Email: evasimone.steinhausen@bg-klinikum-duisburg.de Project Manager: Bernadette Schröder Institute for Medical Informatics, Biometry and Epidemiology/Center for Clinical Studies University Hospital Essen Phone: + 49 201 723–77255 Email: Bernadette.schroeder@uk-essen.de
Role of sponsor {5c}	The PiNGUin study is funded by the G-BA. The treatment costs of the study participants are covered by the statutory health insurance funds. The G-BA commissioned the consortium of the University Hospital Essen AöR and the BG Klinikum Duisburg gGmbH as the independent scientific institution scientifically conducting and analyzing the study.

## Introduction

### Background and rationale {6a}

A non-union is usually defined as a fracture that has not healed after 6 months and is a multifactorial, therapeutically challenging and socio-economically significant complication of fractures. Despite medical advances, non-unions occur in approximately 2–10% of all fractures and can affect up to 30% of patients with certain risk factors [1, 2]. Risk factors include exogenous factors such as open fractures or soft tissue defects and endogenous factors such as obesity, diabetes mellitus or immunosuppression. Often, the exact cause cannot be conclusively clarified. The direct and indirect costs of treatment for non-unions are significantly higher than those associated with regular bone healing [3, 4]. Non-unions often lead to a high level of suffering for patients, as they can cause persistent pain, re-hospitalizations and delayed recovery of work ability.

So far, surgical revision is considered the gold standard for treatment of non-unions. The surgical procedure depends on the type of non-union and usually requires decortication of the non-union and, if necessary, filling of bone defects with autologous/allogenous bone graft or bone graft substitutes. Autologous bone graft is the gold standard, but with the disadvantage of high donor morbidity up to 30%. Typical complications include hematoma, infection and chronic pain at the donor site [5]. Since sufficient stability is essential for bone healing, it may be necessary to extend the osteosynthesis, or even replace the material entirely, depending on the existing osteosynthesis. Various osteosynthesis procedures are available, depending on the location of the non-union. The disadvantages of surgical intervention include re-hospitalization associated with the operation and possible complications such as postoperative infections. Bony consolidation is achieved on average 200 days postoperatively but data on fracture healing rates in non-unions vary. In a review, Gebauer et al. reported fracture-healing rates of 68%−96% after non-union revision, depending on the fracture and surgical procedure and with an average fracture-healing rate of 86% [6].

Treatment with low-intensity pulsed ultrasound (LIPUS) is a possible alternative to surgical revision of non-unions, with skin reactions as an only very rare side effect. An ultrasound signal (mechanical pressure wave) triggers a biological reaction at cellular level. Mechanoreceptors on the cell surface are activated, triggering an intracellular signaling cascade. The cascade increases the upregulation of genes and the expression of proteins and growth factors that are important for bone healing. The inherent therapeutic advantage of this method is the non-invasiveness of the procedure, which is reflected in several patient-relevant endpoints (e.g., surgery-related morbidity, re-hospitalization). In addition, patients can perform the treatment themselves and at home and therefore do not have to be re-hospitalized.

LIPUS treatment has been used for many years and indications include recent fractures for accelerated fracture healing, delayed fracture healing and non-unions [7, 8]. In studies on the treatment of non-unions with LIPUS, promising results were achieved with healing rates of 54–100% and a pooled average healing rate of 88% [6, 9–13].

In their study of 66 patients, Gebauer et al. achieved bony healing in 85% of cases after application of LIPUS [6]. Majeed et al. were able to show a healing rate of 78% in their study conducted on 47 patients with non-unions of the foot and ankle [14]. Bawale et al. achieved a cure rate of 67% in their study of 62 patients [9]. The results were compared with the expected spontaneous cure rate of 0%, leading the authors to conclude that a cure rate of 67% represents convincing evidence of efficacy. In their retrospective study of 767 patients, Zura et al. were able to show a consolidation rate of 86% for non-unions that were older than 12 months [13]. Frankel et al. describe the largest cohort with 4,999 patients and showed consolidation rates of 80–90% [15]. However, in none of the studies mentioned a control group was included.

The stated duration of LIPUS application until fracture consolidation varies in the individual studies [6, 16, 17]. While bony consolidation was achieved after an average of 154 days according to Nolte et al., the study by Gebauer et al. reported an average treatment duration of 168days [6, 17]. The age of the non-union has a significant influence on the duration of treatment as well [16]. In most studies, a maximum treatment duration of 200 days is sufficient and thus corresponds to the healing time after a surgical revision.

With the exception of the studies by Frankel et al. and Zura et al., there are some limitations of studies on non-unions. The number of cases is often small; in some cases, only 14 patients are reported as a case series [12, 13, 15, 18, 19]. In addition, these are exclusively single-arm studies, most of which were conducted retrospectively. The common endpoint of the studies is fracture healing, although this is not uniformly defined. Further clinical endpoints are mentioned in some studies, but again it is also not described how the endpoints were precisely defined, recorded or evaluated. Another shortcoming of the studies to date is the evaluation. In some studies, it is described that patients with insufficient compliance or treatment discontinuation were excluded from the evaluation [9, 15, 17]. As a result, the intention-to-treat principle is violated and the stated healing rates of 88% on average are probably lower. A conservative interpretation of the study results therefore assumes a fracture healing rate of 75% with LIPUS therapy [20]. According to the current literature, the fracture healing rate for treatment with LIPUS is lower than after surgical revision (75% vs. 86%), but still comparably good due to the inherent advantages of the lack of risks. In addition, the costs of LIPUS treatment proved to be significantly lower compared to surgical intervention.

The results to date in the treatment of non-unions with LIPUS are encouraging, however, the reliability of the results is still insufficient due to mentioned shortcomings of the studies.

Beyond this background, the aim of this randomized controlled study is to achieve a sufficiently high level of certainty in the results. If LIPUS treatment is proven not inferior to surgical revision, an alternative therapy will be available in the future and health insurance companies could cover the costs, which is particularly advantageous for high-risk patients.

### Objectives {7}

The study aims to show that in patients with non-unions of the upper or lower extremity, treatment with LIPUS is not inferior to surgical intervention in terms of the fracture-healing rate as primary endpoint (non-inferiority study). Fracture healing is defined as bony consolidation and functional restoration (composite endpoint).

### Trial design {8}

The study is conducted as an open, randomized controlled trial (RCT) with a medical device, designed as a non-inferiority study. Patients with non-union of the upper or lower extremity are randomly allocated in a stratified 1:1 ratio into one of two treatment groups: (1) LIPUS treatment (interventional group); treatment with LIPUS will be self-administered by the patient for 20 min per day. The total duration of treatment should be 200 days. (2) Surgical group (control group); the surgical group will serve as a control group and will receive surgical revision as by standard of care. The duration of the entire study will be five years with a recruitment period of 24 months and a follow-up period per patient of 12 months from the start of the intervention.

## Methods: participants, interventions and outcomes

### Study setting {9}

Overall, 370 patients will be recruited by 20 hospitals in Germany. The study was approved by the Ethics Committee of the Medical Faculty of the University of Duisburg-Essen (24–12,247-BO) and registered at the German Register for Clinical Trials (Deutsches Register Klinischer Studien (DRKS);

DRKS00034357): https://drks.de/search/de/trial/DRKS00034357/details.

For the preparation of the study protocol, the SPIRIT guidelines were implemented [21].

### Eligibility criteria {10}

#### Inclusion criteria

1. Presence of non-union after a fracture of the lower or upper extremity including the clavicle
2. Age of fracture of ≥ 6 months and ≤ 24 months
3. Age of patient ≥ 18 years
4. Primary osteosynthesis of the fracture
5. Bony defect of ≤ 1 cm
6. Closed soft tissues
7. Informed consent dated and signed prior to any study activity in accordance with the International Council for Harmonization of Technical Requirements for Pharmaceuticals for Human Use (ICH)—Guideline for Good Clinical Practice (ICH-GCP Guideline) and local laws
8. Statutory health insurance

#### Exclusion criteria

1. Unstable non-union/osteosynthesis
2. Non-union of the vertebral body or skull
3. Non-union of the small bones of the hand and toes
4. Non-union after osteotomy
5. Presence of a long spiral fracture (length > 3 cm)
6. Pathological fractures due to a malignant tumor or metastasis
7. Surgery performed on the fracture/non-union in the last four months
8. Florid infection/osteomyelitis of non-union, exposed osteosynthesis material, soft tissue defects
9. Therapy with cytostatics
10. Pacemaker
11. Pregnancy/breastfeeding
12. Presence of comorbidity (e.g., psychiatric illness, dementia, excessive drug abuse) which, at the discretion of the treating investigator, may impair the ability to give consent, participation in the study, and compliance.
13. Participation in another interventional study, if this impairs participation in the described study

### Who will take informed consent? {26a}

Prior to any study-related activities, a trained physician of the study team at the respective site will inform the patient about the nature of the study, all related measures and procedures, upcoming (follow-up) visits at the site and risk and benefits. Patients will be given sufficient time to ask questions or to reconsider their participation before giving written informed consent.

### Additional consent provisions for collection and use of participant data and biological specimens {26b}

No additional data or biological samples will be collected from the study patients.

## Interventions

### Explanation for the choice of comparators {6b}

Compared to single-arm studies conducted to date [6, 9–19], we aim to achieve more reliable results by including a control group. Surgical revision is considered as appropriate control intervention as it represents the gold standard in the treatment of non-unions to date. Based on previous studies, a fracture-healing rate of 86% is assumed in the surgical treatment group compared to an assumed healing rate of 75% for patients treated with LIPUS.

### Intervention description {11a}

The intervention in the study group will consist of daily 20-min treatment with LIPUS in the area of non-union using the ultrasound device. Following training in the use of the device, patients can carry out the treatment independently at home. In total, the treatment will last for at least 200 days, of which at least 180 are treatment days. For the use of the ultrasound device, patients attach a strap to the affected limb and place the probe over the marked area of the non-union. The device is equipped with a countdown timer that indicates the end of the treatment with an acoustic signal.

Patients in the control group will undergo surgical revision of the non-union. The type and extent of the operation depend on the fracture and on patient-dependent individual factors and are discussed with the patient. As a rule, the fracture ends need to be resected, the bony defect filled and the osteosynthesis has to be sufficiently stable. Autologous and allogeneic cancellous bone and various bone graft substitutes are available to fill the bony defect, as well as growth factors as additives. Various osteosynthesis procedures are also available for osteosynthesis depending on the fracture type and location (e.g., plate osteosynthesis, intramedullary nail, external fixator).

### Criteria for discontinuing or modifying allocated interventions {11b}

Study participation can be terminated upon the patient’s request, and patients may withdraw from the study for any reason at any time. Investigators may discontinue a patient’s study participation in case of health deterioration, non-compliance, intake of non-permitted medication or if an inclusion or exclusion criterion was violated after study inclusion.

For both groups, after 42 and 120 days of the respective intervention, routine imaging (X-ray or computed tomography, performed independently of the study) will be used to evaluate whether there are any signs of dynamic bone healing. If there are no signs of bone healing at all after 120 days, compared to the initial findings, these circumstances will be discussed with the patient and alternative treatment methods, including a new surgical revision, will be offered. In addition, a wait-and-see approach will be possible at this point. Routine imaging will also be performed after 200 days to confirm that at least three out of four cortices are consolidated. If the non-union is found to be healed after 200 days, no further imaging will be performed unless this is deemed necessary at the doctor's discretion at the end of the study at day 365.

### Strategies to improve adherence to interventions {11c}

Overall, a high level of compliance is expected in this study due to the patients’ long previous history of pain burden. In addition, compliance will be promoted by the fact that LIPUS treatment is pain-free, and the device is small and easy to handle and transport (e.g., also on vacation). In addition, compliance with the daily use of ultrasound treatment will be displayed to patients and investigators via the ultrasound device so that prompt countermeasures will be taken if compliance should be insufficient. This involves reminding the study sites to contact the patients via the central data management system. Lastly, compliance will be promoted by the fact that travel expenses of up to EUR 200 per patient can be reimbursed.

### Relevant concomitant care permitted or prohibited during the trial {11d}

Patients will be informed about which medications or additional interventions they may or may not take in this study. This applies to cytostatic drugs which are an inadmissible concomitant medication in this study as well as therapies other than the intervention mentioned and the surgical treatment described, which are also intended to stimulate bone healing (such as shock waves), and are not permitted during the study. Systemic diseases (e.g., diabetes mellitus) and other medications that may have an influence on bone healing (e.g. non-steroidal anti-inflammatory drugs) will be documented. Accompanying physiotherapy and rehabilitation measures before and during the study to improve the function of the affected extremity will be permitted and desired. Surgical revision for persistent non-union without any improvement in routine imaging should be performed at the earliest four months after the index operation or after the start of treatment with LIPUS. This also corresponds to standard treatment without study participation.

### Provisions for post-trial care {30}

After completion of the study, patients will be allowed to continue LIPUS therapy if consolidation is incomplete but progressing. Patients will be insured as part of the study in case of harm. The insurance will also include a commuting accident insurance.

### Outcomes {12}

#### Primary outcome

The primary outcome will be fracture healing 12 months after the start of treatment, defined that both, bony consolidation and functional restoration, must be present (composite endpoint):

Bony consolidation is defined as at least three out of four cortices are consolidated based on the radiological findings.

Functional recovery will measured using patient reported outcome measures (PROM) separately for the upper and lower extremity: Disability of Arm, Shoulder and Hand (DASH) score with a minimal clinically important difference (MCID) of 10 points and Lower Extremity Functional Scale (LEFS) with an MCID of 9 points. The primary outcome is met, if either the score will be at least as good as the normative value of the healthy population, depending on age and sex, or if at least an improvement equal to the MCID will be achieved for the respective outcome.

##### Disability of Arm, Shoulder and Hand (DASH) questionnaire:

The DASH questionnaire, which has been established for over 25 years, will be used to record functional limitations of the entire upper limbs. Its validity has been demonstrated by several studies [22, 23]. It consists of 30 questions, which are answered using a 5-point Likert scales of different characteristics. The maximum score is 100, with low scores indicating a minor limitation. The first part comprises questions on the functional capacity of the upper extremities, while the second part requires answers to questions on symptoms. The questionnaire was developed by the American Academy of Orthopedic Surgeons (AAOS) in collaboration with the Council of Musculoskeletal Specialty Societies (COMSS) and the Institute for Work and Health (Toronto, Canada). For this study, the validated German version is used [24]. To assess whether functional restoration is present, the DASH must show an improvement of at least 10 points compared to baseline or the score must be at least as good as the normative value of the healthy population. A study on normative values in the German population serves as the basis for the normative values [24]. Here, the median values of the non-clinical manual sample are considered normative values, depending on age and sex [24]. The norm values for a manually active sample are taken into account, since these are somewhat higher than those for a non-manually active sample are. It is considered a success for all patients if they have at least as good a value as the norm value of the healthy, manually active sample, regardless of whether they are engaged in manual activity or not.

##### Lower Extremity Functional Scale (LEFS) questionnaire:

The LEFS is a validated self-assessment questionnaire that records activity restrictions following lower limb injuries and objectively depicts the course of therapy. It consists of 20 questions on various activities, answered using a 5-point Likert scale (“no difficulties”: 4 points to “extreme difficulties”: 0 points). The maximum achievable score is 80, with a higher score indicating better function. In a Dutch study from 2017, a median score of 77 was determined for 1,014 participants, with only slight differences between women and men (median score 76 and 78) [25]. In this study, the validated German version of the questionnaire will be used, for which a high reliability (ICC: 0.98) and consistency (CA: 0.95) was shown [25]. To assess whether functional production is present, the LEFS must either show an improvement of at least 9 points compared to baseline or the score must be at least as good as the normative value of the healthy population. A study by Dingemans et al. on normative values in the Dutch population serves as the basis for the normative values, as there is no study on normative values in the German population. It is assumed that the Dutch normative values are transferable to the German population. The median values of the healthy sample as a function of age and sex are regarded as normative values [24].

#### Secondary outcomes

##### Time to fracture healing (radiological)

Fracture healing will be recorded in the electronic case report form (eCRF) at each visit and time from baseline to healing (in days) will be calculated for this outcome. Fracture healing will be interval-censored data, as it will not be known when fracture healing will have taken place between visits.

##### Time until full weight bearing is achieved

Full weight bearing will be assumed if the patients can bear their own body weight on the affected lower limb without any aids. Full weight bearing of the upper limb will be confirmed if the patient can perform everyday tasks such as carrying a box of water or lifting 10 kg with the affected arm. The assessment will depend on the performance of the unaffected side, as patients' abilities vary greatly and it is up to the investigator to decide whether full weight-bearing capacity of the upper limb has been reached. Weight bearing will be recorded in the eCRF at each visit and the time span (in days) since baseline will be calculated. If the exact date is not known, the time until the first visit with full weight-bearing load will be considered (interval-censored data).

##### Range of motion

The range of motion of the joints adjacent to the non-union will be documented in all planes of motion using the neutral zero method. The survey forms for upper and lower extremities according to the neutral zero method of the German Social Accident Insurance will be used.

##### Prevalence and intensity of pain in the area of non-union

The prevalence and intensity of pain will be assessed using the Numerical Rating Scale (NRS). On a scale from 0 (no pain) to 10 (worst pain imaginable), patients should indicate their current perception of pain at each study visit. The NRS is a recognized tool for assessing pain intensity in medical practice and research and is recommended by the “Initiative on Methods, Measurement and Pain Assessment in Clinical Trials” (IMMPACT). Furthermore, the use of pain medication will be recorded at each visit.

##### Health-related quality of life

Health-related quality of life Quality of life will be assessed using the German validated version of the Short Form 12 (SF-12). The SF-12 is a generic questionnaire and an abbreviated version of the Short Form 36. It consists of 12 questions divided into eight different dimensions that provide information about the patient's health status [26]. It provides information on general health perception (one question), physical health (two questions), impaired physical role functioning (two questions), physical pain (one question), vitality (one question), mental health (two questions), impaired emotional role functioning (two questions) and social functioning (one question). The questionnaire is widely used in clinical research, as it is easy to complete due to its short completion time of approximately 2.5 min.

##### Occurrence of complications/incidents

At each visit, patients will be asked about complications (e.g., secondary dislocations, material fractures, re-fractures, re-hospitalization) or other incidents.

##### Performance and time until possible (re-)operations or (re-)osteosynthesis/incidence and duration of hospitalization

At each visit, patients will be asked whether and when they have had further operations or other hospital stays in the meantime. Since it can be assumed that the re-operation of the non-union is performed at the respective study center, the corresponding data can be found in the hospital information system of the respective center. Surgeries or hospital stays that were performed externally will be queried and the corresponding letters will be requested from the patients. This data will be used to calculate the time in days until the first (re-)operation and the total duration of the hospital stay in days.

##### Duration and reason for incapacity for work

The duration of incapacity for work will be recorded based on the certificate of incapacity for work. As both the duration and the reason for incapacity for work are important for this study in order to differentiate between incapacity for work due to the non-union and other reasons for incapacity for work, the corresponding data from the certificate of incapacity for work (International Statistical Classification of Diseases and Related Health Problems; ICD.10 code) will be collected. If the attending physician at the respective study site did not issue the certificate of incapacity for work, the patient will be asked to present it to the study team. Alternatively, with the patient's consent, the ICD.10 code will be requested from the physician who issued the certificate.

**Fig. 1:**
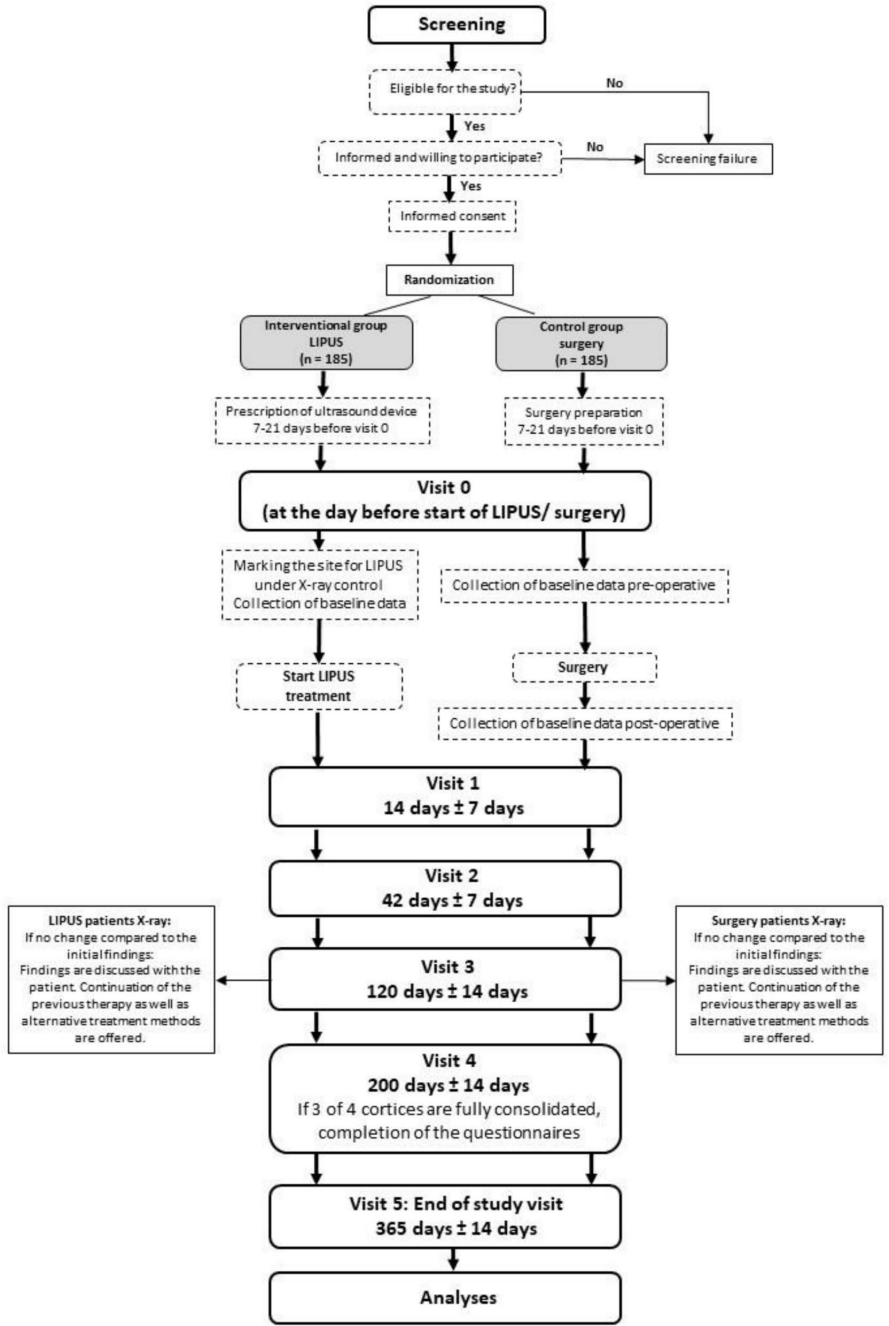
Flow diagram

### Participant timeline {13} (Fig. 1)

Seven visits will take place at the respective study center. During the screening visit, the inclusion and exclusion criteria are checked, and the patient is informed about the study. If these criteria are met, consent is obtained and the patient is randomized into one of the two groups. Patients receiving LIPUS treatment are prescribed an ultrasound device. For patients in the surgical group, preparations for the operation are made (e.g. anesthesia information). After the patient has been randomized, 7–21 days may elapse before the ordered ultrasound device arrives at the study site, or before all preparations for surgery have been completed and a date has been set. If more than four weeks elapse between randomization and the start of ultrasound therapy or surgery, the inclusion and exclusion criteria must be checked again at the baseline visit for possible changes. Repeated imaging is at the discretion of the attending physician and is performed independently of the study.

**Fig. 2:**
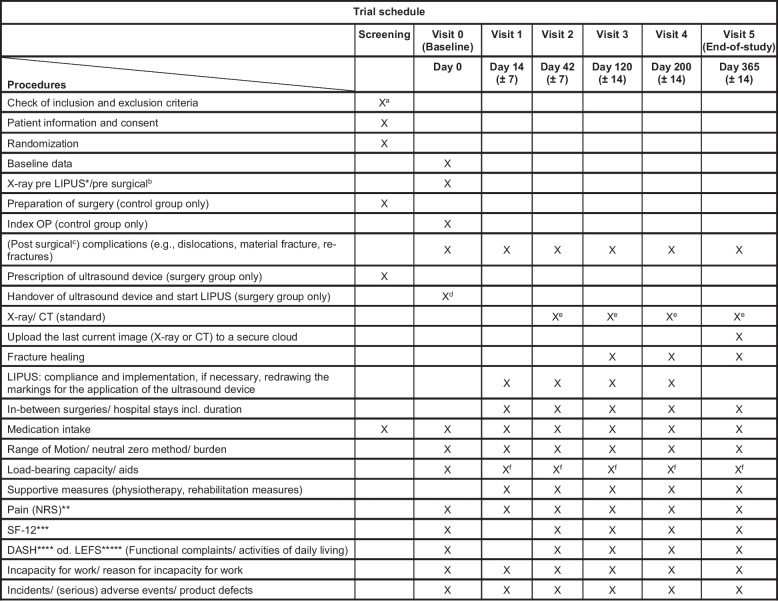
Trial schedule. *low-intensity pulsed ultrasound. **Numeric Rating Scale. ***Short Form-12. ****Disabilities of the Arm, Shoulder and Hand Questionnaire. *****Lower Extremity Functional Scale Questionnaire. ^a^If more than 4 weeks pass between screening and visit 0, the inclusion/exclusion criteria will be checked again and, at the discretion of the attending investigator, imaging will be repeated if necessary. ^b^X-ray/CT control for surgical planning (standard examination; may not be necessary if current imaging will be available that is sufficient for surgical planning. This will be at the discretion of the attending investigator). ^c^Postoperative complications that are attributable only to the surgery (e.g., wound infection) will be recorded accordingly only for the control group. ^d^Before starting the intervention, the correct location for LIPUS will be marked under X-ray control. ^e^If the bone is found to be consolidated (at least 3 out of 4 cortices will be radiologically confirmed to be consolidated) at visit 4, no further imaging will be performed unless this would be necessary at the investigator`s discretion. ^f^If full-weight bearing is possible, the time at which full weightbearing is reached will be documented

At the baseline visit (day 0), patients in the intervention group will receive the ultrasound device and will be instructed on the use. In addition, the site of the non-union to be treated by ultrasound will be marked. Before the start of LIPUS treatment or surgery, baseline data will be collected (Fig. 2) and a physical examination will be performed. In both treatment groups, NRS will be used to evaluate the patients’ pain, and questionnaires will be used to assess the general quality of life (SF-12) and the function of the affected limb (DASH or LEFS).

Visit 1 will take place as the first check-up 14 days after the start of the ultrasound treatment or after the operation. A physical examination will be carried out again and the range of motion, the weight-bearing capacity and the NRS will be documented. Patients will be asked about aids, supportive measures, medication taken, possible complications from the treatment and (serious) adverse events ((S)AEs) and (serious) adverse device effects ((S)ADEs) will be recorded.

Visits 2 to 4 will take place at around 6 weeks, 4 months and 6 months after baseline. Visit 5, which will take place 1 year after baseline, is the final visit. The dates correspond to the standard follow-up visits carried out during the treatment of non-unions. A physical examination will be carried out at all appointments, and the corresponding questionnaires will be given to the patient. All complications that will have occurred during treatment will be assessed. An X-ray or CT scan will be performed at the discretion of the investigator in accordance with routine treatment (Fig. 2).

### Sample size {14}

Sample size estimation is based on a Z-test with non-pooled variances to verify the non-inferiority of treatment with LIPUS compared to surgical treatment. A fracture-healing rate of 86% is assumed in the surgical treatment group based on previous studies [6]. In the intervention group, a fracture healing rate of 75% is assumed [6, 9–14]. Since spontaneous healing, i.e. healing without treatment, only occurs in an estimated 5% of cases, it can be assumed that both treatments are superior to no treatment.

LIPUS treatment is assumed non-inferior to surgical treatment if the proportion of fracture healings in the intervention treatment group is less than 25 percentage points lower than in the control group. If a fracture-healing rate of 86% is assumed with surgical treatment and LIPUS treatment is actually non-inferior to surgical treatment, this means that fracture healing can be expected in more than 61% of those treated with LIPUS. In these, surgical intervention can be avoided and all the risks associated with it (e.g., re-hospitalization, secondary diseases or side effects of surgery such as infections and anesthetic incidents). Patients, whose fractures have not healed, will still be able to undergo surgical intervention at a later date. Assuming healing rates of 86% for surgical and 75% for LIPUS treatment, patients with incomplete fracture healing would be considered as treatment failures, included in the 14% or 25% unsuccessful treatment outcomes. Since no relevant side effects are to be expected from LIPUS treatment, the risk of delayed fracture-healing in the event of unsuccessful LIPUS treatment is therefore offset by the risk of unnecessary surgical intervention. The non-inferiority threshold of −25 percentage points is considered appropriate in this context.

With the above assumptions, a one-sided significance level of 2.5% and a power of 90%, 166 patients are required per group. In addition, a dropout rate of 10% is assumed. The proportion of dropouts of max. 10% appears realistic, as patients with non-unions require regular clinical and radiological check-ups (timed to coincide with the study visits) even without participating in the study. The intention-to-treat (ITT) analysis will be used to include patients who will not comply with the protocol. Based on the above assumptions, 184 patients will have to be recruited per treatment group. Accordingly, a total number of 370 cases will be required.

### Recruitment {15}

As part of the study preparations, the study sites were asked in advance to estimate the number of possible patients in order to be able to assess whether a sufficient number of patients could be realized with the 20 study sites. Recruitment will be checked in real time by the central data management of the Center for Clinical Trials Essen (ZKSE). The project management will be in close contact with the sites in order to resolve possible difficulties in recruiting patients at an early stage through regular communication with the study teams. The sites will also be supported centrally and locally by a monitor.

## Assignment of interventions: allocation

### Sequence generation {16a}

Randomization is carried out using the software Clincase developed by Quadratek (Berlin). In order to obtain comparable groups, randomization will be stratified by upper or lower extremity, age (≤ 60 or > 60 years) and sex in order to achieve balanced factor distributions for these relevant predictors. Other variables that could have an important influence on the primary outcome are assumed to be evenly distributed across the treatment arms by randomization. Each randomized patient will be part of the ITT analysis.

### Concealment mechanism {16b}

Considering the given strata information, the study team will perform the randomization by use of the eCRF. The system will provide a randomization code based on a randomization list and will also record the respective result. The system will provide a randomization code based on a randomization list and will also record the respective result. Only authorized people of the ZKSE and the eCRF provider will have access to the randomization list.

### Implementation {16c}

The randomization-ID list will be created by an independent statistician and will be stored in the randomization software. Randomization will be performed dynamically based on the minimization procedure. The information regarding the allocation of patients to the corresponding group will be generated by the software system and automatically communicated to the study site.

## Assignment of interventions: blinding

### Who will be blinded {17a}

PiNGUin is designed as an open-labelled study, as blinding of patients and treating investigators is not possible due to the use of the ultrasound device or the implementation of surgery. A possible bias will be mitigated by the blinded endpoint committee. To assess the primary endpoint, the last available X-ray/CT image of each patient will be presented to the independent endpoint committee. This means that the assessor will not be able to draw any conclusions as to whether, for example, implants have been replaced. As a result, it will be more difficult for the reviewer to assign the patients to the respective arm, which mitigates a possible subjective bias towards one type of treatment.

### Procedure for unblinding if needed {17b}

Not applicable, as the investigators and patients will not be blinded.

## Data collection and management

### Plans for assessment and collection of outcomes {18a}

Data will be derived from paper-based or electronic patient records, depending on the systems used by the respective study site. Data will be collected via the electronic data capture software Clincase (Quadratek Data Solutions Ltd., Berlin). The software has an integrated audit trail and is a GCP-compliant data management system. All data management will be carried out in accordance with harmonized standard operating procedures (SOPs). As part of the data collection at the centers, the ZKSE will provide predefined communication channels to give the centers the opportunity to raise questions or concerns. Source data will be reviewed by the monitor during on-site visits.

### Plans to promote participant retention and complete follow-up {18b}

If a patient does not appear to a planned study visit, the respective study personnel will attempt to contact the patient and reschedule the missed visit as soon as possible. The patient will be informed about the importance of keeping the assigned appointment and it will be ensured whether the patient wants and/or should continue in the study. Before a patient will be classified as “lost to follow-up”, the investigator or a member of the study team must make every effort to re-establish contact with the participant (three phone calls if possible and, if necessary, writing to the participant's last known mailing address). These attempts at contact will be documented in the participant's medical record. Data collected for the patients up to the time of withdrawal from the study will be used for analysis.

### Data management {19}

The ZKSE will take technical and organizational measures that meet the requirements of the relevant data protection regulations in order to adequately protect the data from misuse and loss. All electronic data will be stored on physical or virtual servers at Essen University Hospital. Study data of the study sites will be collected via the web-based electronic data collection system (EDS) software Clincase. The primary storage of these data will take place on servers in two Quadratek data centers in Berlin. After the database will be closed, the data will be finally exported and transferred to the servers of the University Hospital Essen and the data in the Quadratek data centers will then be deleted. Storage of data on local PCs of the ZKSE is not permitted. The computers used for the project are protected against external access by a firewall configured by the hospital computer center of Essen University Hospital. User management is implemented under Windows. Remote access to the data is excluded. Only the people involved in the project will have access to the data. The ZKSE, as an independent institution from the other study personnel, will be responsible for data quality management and monitoring of this study. Data will be checked for missing data, data out of range, inconsistent or potentially incorrect by date management. For this purpose, checks such as ranges, date inconsistency or presence/absence of data or complex cross-form checks such as date discrepancies between visits will be carried out. Specific accuracy and reliability checks will be performed automatically while the forms in the software are completed to enable immediate quality control. Data management will perform further manual checks for completeness and plausibility of the study data and will clarify any question with the study centers electronically via the software. Further checks for plausibility, consistency and completeness of the data will be carried out after completion of the study. An audit trail will ensure the traceability of all changes made in the database. If no further corrections are to be made, the database will be closed and the final export will be used for the statistical analysis. The pseudonymized clinical data will be managed and analyzed at the University Hospital Essen, Institute for Medical Informatics, Biometry and Epidemiology/Center for Clinical Trials Essen as part of data management and statistical evaluation. Only the project staff will have access to the project-specific data in accordance with the rights assigned to them personally. All data manipulations will be documented in log files, which will contain the type of manipulation, the time and the user ID. These log files will be protected against unauthorized access and will only be accessible to administrators in an emergency.

### Confidentiality {27}

The study will be conducted in accordance with the GCP guidelines and the Declaration of Helsinki. Data protection will be ensured in all phases of data collection, transmission, processing and analysis in accordance with the laws and regulations of the Federal Republic of Germany and international standards. The integrity, confidentiality and availability of personal data will be ensured in accordance with the General Data Protection Regulation (GDPR). The principle of purpose limitation of data collection, transmission and evaluation will be observed and the confidentiality of the data will be guaranteed both for the employees and for the subcontractors/data processors. Confidentiality of study patients will be ensured by pseudonymization of patient data in the EDC system. The data collected will be deleted no later than 10 years after the end of the clinical study ((EU) 2017/745, Annex XV, Chapter III, Sect. 3).

### Plans for collection, laboratory evaluation and storage of biological specimens for genetic or molecular analysis in this trial/future use {33}

Not applicable as no laboratory evaluation or storage of biological specimens will be carried out in this study.

## Statistical methods

### Statistical methods for primary and secondary outcomes {20a}

Treatment with LIPUS will be analyzed with a non-inferiority test against surgical treatment for the primary endpoint of fracture healing. A one-sided 97.5% confidence interval will be estimated for the difference in the proportion of fracture healing between the two groups. If the lower limit of the confidence interval will be greater than the non-inferiority limit of −25 percentage points, the null hypothesis of no non-inferiority will be rejected at the significance level α = 0.025. In addition, a generalized linear mixed model (GLMM) will be fitted to evaluate the robustness of the result for the binary endpoint of fracture healing. Treatment, sex, age and upper vs. lower limb will be considered as fixed effects. The study sites will be modeled as random effects. In order to estimate the risk difference for the treatment in the GLMM, the identity link will be considered as a link function for this endpoint. In addition, the analyses will be performed separately for radiographic bony consolidation and functional restoration. An intention-to-treat (ITT) analysis will be performed as well as a per-protocol (PP) analysis as a sensitivity analysis. The safety analysis will be based on the documented (S)AEs/(S)ADEs. All secondary endpoints will be analyzed exploratively, that is, without adjustment for multiple testing, using standard methods of statistical inference appropriate for the scale level of the endpoint in question. Scores for DASH and LEFS will be considered as continuous outcomes for which linear mixed regression models will be used, where sex, age, and upper or lower extremity will be included as fixed and study sites as random effects.

### Interim analyses {21b}

Interim analyses will only be conducted for the purpose of evaluating safety data and making possible recommendations for adjusting the risk–benefit assessment. If serious deviations from the protocol should be identified, the study will be prematurely terminated at the respective study site. The principal investigator of the study will have the right to terminate the entire study for ethical, clinical, or administrative reasons or the responsible ethics committee may recommend it, the scientific/ethical review committee accompanying/supervising the study, or a state monitoring institution. If new findings and/or risks come to light that necessitate a new risk–benefit assessment and the outcome is unfavorable, this will be a reason for terminating the entire study. The termination of the study would be communicated to the responsible ethics committees. If the study is terminated prematurely, all investigators and participating patients will be informed. The investigators at the study site will also ensure that appropriate further treatment and follow-up will be organized for the recruited patients. A follow-up appointment will be arranged for all patients within 14 days. If further follow-up care is required, this will be organized accordingly by the investigators of the study site.

### Methods for additional analyses (e.g., subgroup analyses) {20b}

Subgroup analyses will be performed separately for upper and lower extremities, age (≤ 60 vs. > 60 years) as well as for sex.

### Methods in analysis to handle protocol non-adherence and any statistical methods to handle missing data {20c}

At visit three, approximately 120 days after start of the intervention, it will be established whether the treatment has been successful. If the treatment is classified as unsuccessful for patients, the corresponding patients from both treatment groups will be offered alternative or further treatments, including (renewed) surgery. Should such additional interventions occur after visit 3, the data from visits 4 and 5 should only be viewed with caution. If analyzed according to the ITT principle, positive treatment effects of the surgical intervention could overestimate the effect of the LIPUS treatment. If patients with additional surgical intervention are counted as surgically treated, the effect of LIPUS treatment could also be overestimated, as the patients who failed and therefore underwent surgery are no longer considered in this group. If all patients with an additional procedure were excluded from the analysis, the treatment effects in both groups could be overestimated, as these patients with a failure are no longer included. For the analysis of the primary endpoint, all patients who will receive an additional surgical intervention are therefore classified as failures, as it can be assumed that the intervention was performed due to a lack of success. For the evaluation of the secondary endpoints, the last observed values before an additional surgical intervention will be carried forward (last observation carried forward (LOCF)). Because of the different interventions (non-recurring surgery vs. daily treatment with LIPUS), a smaller compliance rate in the LIPUS group will be expected, resulting in presumably lower success rates. To account for these compliance differences in the application of the treatments, the ITT population will be regarded for primary analyses. In order to estimate the differences of the treatments when applied correctly, the PP population will be analyzed additionally.

If less than 5% of the values of the primary endpoint are missing, the primary analysis will be performed on the complete data available and a sensitivity analysis will be performed using LOCF. If at least 5% of the values are missing, the primary analysis will be performed on the basis of multiple imputation using the MAR (missing at random) assumption. The analysis on the complete data will be performed as a sensitivity analysis. Further details on the planned analyses of all endpoints and on how to deal with missing values will be defined in the statistical analysis plan.

### Plans to give access to the full protocol, participant level-data and statistical code {31c}

Upon publication of the planned research papers, we intend to share the de-identified data for future research purposes upon reasonable requests.

## Oversight and monitoring

### Composition of the coordinating centre and trial steering committee {5d}

The national principal investigator and her deputy will lead the study. The Institute for Medical Informatics, Biometry and Epidemiology/Center for Clinical Trials Essen is responsible for clinical project management, data management, monitoring and statistical analysis and acts as an independent scientific institution. The study will be monitored by the G-BA with regard to the applicable guidelines.

### Composition of the data monitoring committee, its role and reporting structure {21a}

To ensure patient safety, a Data Safety Monitoring Board (DSMB), consisting of three experts (radiologist, statistician, trauma surgeon), will supervise the study. This committee will hold biannual meetings and will monitor the progress of the study in terms of recruitment and conduct, evaluate the safety data based on interim analyses and provide recommendations to adjust the risk–benefit assessment.

### Adverse event reporting and harms {22}

(Serious) adverse events ((S)AEs) and (serious) adverse device effects ((S)ADEs) will be documented in the eCRF at each study visit or as soon as they become known to the investigator and reported to the ZKSE within 7 working days (AEs/ADEs) or 24 h (SAEs/SADEs) of becoming known. An ADE is defined as an AE related to the use of the ultrasound device and includes events resulting from inaccurate instructions, malfunction or user error. An SADE is defined as a defect that has resulted in any of the characteristics of an SAE, i.e., death, life-threatening, requiring hospitalization or prolonging an existing hospitalization, or resulting in prolonged significant disability or incapacity.

An incident is defined as a malfunction or deterioration in the characteristics or performance of the ultrasound device, including application errors due to ergonomic features, as well as an inadequacy of the information provided by the manufacturer or an undesirable side effect. Incidents do not include individual anatomical or physiological characteristics of the patient or human errors (e.g., due to the influence of alcohol or drugs, fatigue, lack of device instruction) that have led to a treatment problem without a defect or malfunction of a medical device. For the CE-certified ultrasound device that is already on the European Union market, any incident related to the device that is not an expected side effect, clearly documented in the product information, will be reported by the manufacturer to the Federal Institute for Drugs and Medical Devices via an incident report.

### Frequency and plans for auditing trial conduct {23}

The investigators and institutions involved in the study allow study-related monitoring and audits by the G-BA. In the event of an audit, the investigator will agree to grant the auditor access to all study-related documents. This will also apply to all local or state authorities that are legitimized by applicable law.

### Plans for communicating important protocol amendments to relevant parties (e.g., trial participants, ethical committees) {25}

All changes to the protocol are submitted to the relevant ethics committees for approval and will be entered into the study registry. Already randomized patients will have to sign an updated informed consent form. All deviations from the protocol will be fully documented and tracked using breach reporting forms to ensure the transparency and integrity of the protocol.

### Dissemination plans {31a}

After completion of the study, the study management will submit the final report to all designated recipients within the applicable deadlines. The final report will be prepared in accordance with the ICH E3 guideline and the G-BA's specifications and will be submitted to the G-BA together with the statistical analysis plan. Publication of the results will be independent of the outcome. The results will be published in peer-reviewed international medical journals and presented at national and international conferences.

## Discussion

The aim of this G-BA-funded study is to prove that in patients with non-unions, treatment with low-intensity pulsed ultrasound is not inferior to surgical intervention in terms of the fracture healing rate endpoint (non-inferiority question). Despite medical advances, non-unions occur in approximately 2–10% of all fractures and can affect up to 30% if certain risk factors are involved [1, 2]. To date, surgical revision has been the gold standard in the treatment non-union, with the surgical procedure depending on the type of non-union. The disadvantages of surgical intervention are re-hospitalizations associated with the operation as well as possible secondary diseases and side effects such as postoperative infections, anesthetic incidents and embolisms. A possible alternative to surgical revision for non-unions is treatment with LIPUS, although this is currently considered an individual health service and the benefits over surgery have not yet been shown in a comparative study with sufficient certainty of results.

One of the strengths of our study lies in its design. The study will be conducted as an open, controlled and randomized study with 370 patients over 2 years at approximately 20 study sites. Blinding of patients and investigators is not possible due to the nature of the intervention. A possible bias will be significantly attenuated by the choice of an endpoint committee. To assess the primary endpoint (assessment of fracture healing), only the last available X-ray/CT image of the individual patients will be presented to the independent endpoint committee. This means that the assessor will not be able to draw any conclusions as to whether, for example, implants have been replaced, i.e. whether a surgery has taken place.

To improve the accuracy and reliability of the study, a approved and CE-marked ultrasound device will be used, which has been used for the treatment of non-unions for several years. Furthermore, the device allows compliance control in the implementation of the treatment for the individual patients. In addition, the SF-12, DASH and LEFS questionnaires are widely used and validated. If non-inferiority of LIPUS compared to standard therapy (surgical revision) is proven in this study, patients with non-unions will have a choice of both invasive and non-invasive/non-surgical therapy methods in the future. The costs for LIPUS treatment should also be covered by health insurance companies in the future if non-inferiority is proven. This would be particularly beneficial for patients with other pre-existing conditions and with an increased risk of anesthesia, as well as for patients who refuse surgery for other reasons.

## Data Availability

The Principal investigator, the Project Manager and Data Management will have full access to the data with a personal confidential password. The University Hospital Essen and the BG Klinikum Duisburg own all research data collected as part of this project. The principal investigator takes responsibility for all data collection, management, and sharing of research data. It will be possible to re-use the data for further research, document the validity of the research, and make possibilities for data sharing and collaborations upon reasonable request.

## Abbreviations

CT: Computer tomography
DASH: Disabilities of Arm, Shoulder and Hand Questionnaire
DSMB: Data Safety Monitoring Board
eCRF: Electronic Case Report Form
EDC: Electronic data capture
GCP: Good Clinical Practice
GLMM: Generalized linear mixed model
ICD-Code: International Statistical Classification of Diseases and Related Health Problems
ICH: International Council for Harmonization of Technical Requirements for Pharmaceuticals for Human Use
LEFS: Lower Extremity Functional Scale
LOCF: Last observation carried forward
MCID: Minimal Clinically Important Difference
LIPUS: Low-intensity pulsed ultrasound
NRS: Numerical Rating Scale
PROM: Patient Reported Outcome Measure
SF-12: Short-Form 12
(S)ADE: (Serious) Adverse Device Effect
(S)AE: (Serious) Adverse Event
ZKSE: Zentrum für klinische Studien Essen (Center for Clinical Trials in Essen)

## References

[1] Biberthaler PvG, Martijn. Knochendefekte und Pseudarthrosen. Peter Biberthaler MvG, editor: Springer Berlin, Heidelberg; 2017.

[2] Reeh FM, Sachse S, Wedekind L, Hofmann GO, Lenz M. Nonunions and Their Operative Treatment—a DRG-Based Epidemiological Analysis for the Years 2007–2019 in Germany. Dtsch Arztebl Int. 2022;119(50):869–75. 36352531 10.3238/arztebl.m2022.0300 PMC9989962

[3] Ekegren CL, Edwards ER, de Steiger R, Gabbe BJ. Incidence, costs and predictors of non-union, delayed union and mal-union following long bone fracture. Int J Environ Res Public Health. 2018. 10.3390/ijerph15122845. 30551632 10.3390/ijerph15122845 PMC6313538

[4] Hak DJ, Fitzpatrick D, Bishop JA, Marsh JL, Tilp S, Schnettler R, et al. Delayed union and nonunions: epidemiology, clinical issues, and financial aspects. Injury. 2014;45(Suppl 2):S3-7. 24857025 10.1016/j.injury.2014.04.002

[5] Sen MK, Miclau T. Autologous iliac crest bone graft: should it still be the gold standard for treating nonunions? Injury. 2007;38(Suppl 1):S75-80. 17383488 10.1016/j.injury.2007.02.012

[6] Gebauer D, Mayr E, Orthner E, Ryaby JP. Low-intensity pulsed ultrasound: effects on nonunions. Ultrasound Med Biol. 2005;31(10):1391–402. 16223643 10.1016/j.ultrasmedbio.2005.06.002

[7] Romano CL, Romano D, Logoluso N. Low-intensity pulsed ultrasound for the treatment of bone delayed union or nonunion: a review. Ultrasound Med Biol. 2009;35(4):529–36. 19097683 10.1016/j.ultrasmedbio.2008.09.029

[8] Roussignol X, Currey C, Duparc F, Dujardin F. Indications and results for the Exogen™ ultrasound system in the management of non-union: a 59-case pilot study. Orthop Traumatol Surg Res. 2012;98(2):206–13. 22424956 10.1016/j.otsr.2011.10.011

[9] Bawale R, Segmeister M, Sinha S, Shariff S, Singh B. Experience of an isolated use of low-intensity pulsed ultrasound therapy on fracture healing in established non-unions: a prospective case series. J Ultrasound. 2021;24(3):249–52. 32356220 10.1007/s40477-020-00464-9 PMC8363719

[10] Elvey MH, Miller R, Khor KS, Protopapa E, Horwitz MD, Hunter AR. The use of low-intensity pulsed ultrasound in hand and wrist nonunions. J Plast Surg Hand Surg. 2020;54(2):101–6. 31771389 10.1080/2000656X.2019.1693393

[11] Mayr E, Frankel V, Rüter A. Ultrasound–an alternative healing method for nonunions? Arch Orthop Trauma Surg. 2000;120(1–2):1–8. 10653095 10.1007/pl00021234

[12] Pigozzi F, Moneta MR, Giombini A, Giannini S, Di Cesare A, Fagnani F, et al. Low-intensity pulsed ultrasound in the conservative treatment of pseudoarthrosis. J Sports Med Phys Fitness. 2004;44(2):173–8. 15470315

[13] Zura R, Della Rocca GJ, Mehta S, Harrison A, Brodie C, Jones J, et al. Treatment of chronic (>1 year) fracture nonunion: heal rate in a cohort of 767 patients treated with low-intensity pulsed ultrasound (LIPUS). Injury. 2015;46(10):2036–41. 26052056 10.1016/j.injury.2015.05.042

[14] Majeed H, Karim T, Davenport J, Karski M, Smith R, Clough TM. Clinical and patient-reported outcomes following low intensity pulsed ultrasound (LIPUS, Exogen) for established post-traumatic and post-surgical nonunion in the foot and ankle. Foot Ankle Surg. 2020;26(4):405–11. 31142440 10.1016/j.fas.2019.05.009

[15] Frankel VH, Mizuho K. Management of non-union with pulsed low-intensity ultrasound therapy–international results. Surg Technol Int. 2002;10:195–200. 12384881

[16] Harrison A, Lin S, Pounder N, Mikuni-Takagaki Y. Mode & mechanism of low intensity pulsed ultrasound (LIPUS) in fracture repair. Ultrasonics. 2016;70:45–52. 27130989 10.1016/j.ultras.2016.03.016

[17] Nolte PA, van der Krans A, Patka P, Janssen IM, Ryaby JP, Albers GH. Low-intensity pulsed ultrasound in the treatment of nonunions. J Trauma. 2001;51(4):693–702; discussion -3. 10.1097/00005373-200110000-00012 11586161

[18] Hemery X, Ohl X, Saddiki R, Barresi L, Dehoux E. Low-intensity pulsed ultrasound for non-union treatment: a 14-case series evaluation. Orthop Traumatol Surg Res. 2011;97(1):51–7. 21269906 10.1016/j.otsr.2010.09.016

[19] Lerner A, Stein H, Soudry M. Compound high-energy limb fractures with delayed union: our experience with adjuvant ultrasound stimulation (exogen). Ultrasonics. 2004;42(1–9):915–7. 15047406 10.1016/j.ultras.2003.11.014

[20] Institut für Qualität und Wirtschaftlichkeit im Gesundheitswesen I. Niedrigdosierter gepulster Ultraschall zur Behandlung von Pseudarthrosen. Evaluation Report. Köln; 2022. Report No.: No. 1548.

[21] Chan AW, Tetzlaff JM, Gøtzsche PC, Altman DG, Mann H, Berlin JA, et al. SPIRIT 2013 explanation and elaboration: guidance for protocols of clinical trials. BMJ. 2013;346:e7586. 23303884 10.1136/bmj.e7586 PMC3541470

[22] Beaton DE, Katz JN, Fossel AH, Wright JG, Tarasuk V, Bombardier C. Measuring the whole or the parts? Validity, reliability, and responsiveness of the Disabilities of the Arm, Shoulder and Hand outcome measure in different regions of the upper extremity. J Hand Ther. 2001;14(2):128–46. 11382253

[23] SooHoo NF, McDonald AP, Seiler JG 3rd, McGillivary GR. Evaluation of the construct validity of the DASH questionnaire by correlation to the SF-36. J Hand Surg Am. 2002;27(3):537–41. 12015732 10.1053/jhsu.2002.32964

[24] Jester A, Harth A, Rauch J, Germann G. DASH data of non-clinical versus clinical groups of persons–a comparative study of T-norms for clinical use. Handchir Mikrochir Plast Chir. 2010;42(1):55–64. 20205068 10.1055/s-0030-1247500

[25] Dingemans SA, Kleipool SC, Mulders MAM, Winkelhagen J, Schep NWL, Goslings JC, et al. Normative data for the lower extremity functional scale (LEFS). Acta Orthop. 2017;88(4):422–6. 28350206 10.1080/17453674.2017.1309886 PMC5499335

[26] Wirtz MA. Normierung des SF-12 Version 2.0 zur Messung der gesundheitsbezogenen Lebensqualität in einer deutschen bevölkerungsrepräsentativen Stichprobe. Diagnostica. 2018. 10.1026/0012-1924/a000205.

